# Supplementary material for: Organ Mapping Antibody Panels: a community resource for standardized multiplexed tissue imaging
Source: Nat Methods. 2023 Jul 19;20(8):1174–8. doi: 10.1038/s41592-023-01846-7 (PMC10406602; doi:10.1038/s41592-023-01846-7)
Supplement: Supplementary file 1 — Supplementary Tables 1–3 and figure legends. [file 41592_2023_1846_MOESM1_ESM.pdf]

nature methods

Brief Communication https://doi.org/10.1038/s41592-023-01846-7

Organ Mapping Antibody Panels: a community resource for standardized multiplexed tissue imaging

In the format provided by the authors and unedited

nature methods

Brief Communication

https://doi.org/10.1038/s41592-023-01846-7

Organ Mapping Antibody Panels: a community resource for standardized multiplexed tissue imaging

In the format provided by the authors and unedited

SUPPLEMENTARY INFORMATION

Supplementary Table 1. List of community validated antibodies aggregated across seven OMAPs designed for different highly multiplexed imaging platforms*. Provided as Excel file.*

Supplementary Table 2. Summary of the anatomical structures and cell types identified by each OMAP with core protein biomarkers highlighted*. Provided as Excel file.*

Supplementary Table 3. Antibody Validation Report with example text and required data …….……..……..………………………………..………………..…………………..………….. 4

Supplementary Table 4. Metadata schema for OMAP Table columns ..………..…..………. 6

Supplementary Table 5. Information to include in the OMAP Description Document with example text …..…………………………………………………...………………………… 8

Supplementary Table 1. List of community validated antibodies aggregated across seven OMAPs designed for different highly multiplexed imaging platforms.

Provided as Excel file.

OMAPs from the Human Reference Atlas release v1.3 were downloaded from the Human Reference Atlas Portal (https://humanatlas.io/omap) and antibodies from all seven tables (intestine, kidney, liver, lymph node, lung, pancreas, and skin) were combined into one table ('Combined' tab). Columns for OMAP organ and ID number ('omap') were added to track the source of each antibody. The 'Summary' tab contains four pivot tables that display shared protein biomarkers and antibody clones across OMAPs. Secondary antibodies were omitted from both 'Combined' and 'Summary' tabs.

Supplementary Table 2. Summary of the anatomical structures and cell types identified by each OMAP with core protein biomarkers highlighted.

Provided as Excel file.

Anatomical structures and cell types were exported from the Human Reference Atlas release v1.2 of the relevant organ tables from the ASCT+B Reporter. OMAP authors identified the major anatomical structures defined by their OMAP using knowledge of their respective organ with additional input from experienced pathologists. Some entries were removed or merged to avoid redundancy and increase readability. Cell types were defined by highlighting the single protein biomarker used to best characterize a given cell type (bolded) with additional markers listed. Morphology and spatial location were also used for cell phenotyping as highlighted in Figure 1d. Cell ontology terms were included based on details from corresponding ASCT+B tables and cell ontology databases (EMBL-EBI, https://www.ebi.ac.uk/ols/index; LungMAP, https://www.lungmap.net/explore-data/ontology-browser/).

Supplementary Table 3. Antibody Validation Report with example text and required data.

Components making up an Antibody Validation Report (AVR). Required fields are listed in **bold text**. For additional details, please see the standard operating procedure on authoring AVRs: https://zenodo.org/record/7418624#.Y78RWnbMIuW.

AVR section	Field name	Example
Target information	**Target name**	Cellular tumor antigen p53
Target information	**HGNC ID**	HGNC:11998
Target information	**UniProt accession number** (reference multiple if applicable)	P04637
Antibody information	**RRID**	AB_2206626
Antibody information	**Host**	Mouse
Antibody information	**Isotype**	IgG2b
Antibody information	**Clonality**	DO-7
Antibody information	**Vendor**	Dako
Antibody information	**Catalog number**	M7001
Antibody information	**Recombinant** (Y/N)	N
Antibody information	**Tissue preservation method**	FFPE
Antibody information	**Organ or tissue used for validation**	Skin
Antibody information	**Organ or tissue ontology ID**	UBERON:0002113
Antibody information	**Antibody-based imaging method**	Cell DIVE
Antibody information	**Conjugate**	Cy5
Antibody information	**Author ORCID**	0000-0001-7524-8260
Antibody information	OMAP ID	OMAP-4
Antibody information	Lot number	20082640
Antibody information	Antigen retrieval details	pH 6, pH 9
Antibody information	Manuscript citation DOI	https://dx.doi.org/10.1101/2022.03.30.486438
Antibody information	Validation protocol DOI	https://dx.doi.org/10.17504/protocols.io.bpyxmpxn
Exemplary image of validated antibody	Image, antibody RRID, and brief caption	Caption: Cell DIVE image of p53 (red) and DAPI (blue) in human skin. Expected nuclear colocalization observed with p53 antibody.
Validation data (vendor)	Data sheet URL	Link
Validation data (vendor)	Date accessed	8/29/2022
Validation data (vendor)	Vendor suggested use	SDS-PAGE, WB, IHC

AVR section	Field name	Example
Validation data (laboratory)	Controls used – check those that apply: positive/negative control tissues; isotype control; peptide block; phosphatase treatment; cell line controls; other (please list).	Positive/negative control tissues: Multi tissue TMA including skin and 12 other organs, normal and cancerous. Isotype control: checked.
Validation data (laboratory)	Other antibodies tested – provide vendor and catalog number, clonality, and whether it is a backup clone or not recommended.	Thermo MS-738-PABX (clone DO-7 + BP53-12); not recommended, high background.
Supplemental data	May include example images evaluating several different antibody clones and/or formats for their performance in serial tissue sections with accompanying images as primary characterization data. Additional data may include representative images detailing the impact of cycle number on antibody performance and/or direct and indirect detection of validated antibodies.	

Supplementary Table 4. Metadata schema for OMAP Table columns.

Instructions on standardized formatting and examples for authors submitting an OMAP.

Field name in OMAP table	Description	Required format	Examples
uniprot_accession_number	Identifies the target protein (see https://www.uniprot.org). For human protein panels, be certain to include human protein designation. If UniProt ID cannot be found for the antibody, please leave the field blank.	Alphanumeric (more info here); for multiclonal (pan-) antibodies, list IDs for all targeted proteins as a comma delimited list.	A2BC19, P12345, Q9BZS1
HGNC_ID	Gene identification number from Human Gene Ontology Nomenclature Committee (HGNC) encoding the target protein (see https://www.genenames.org/).	HGNC:####	HGNC:4947, HGNC:3612
target_name	Provides a common name for the target protein being detected by assay. Please spell out Greek letters (alpha, beta, gamma, delta, etc).	Commonly used name or protein abbreviation.	CD20, ICAM, Somatostatin, Alpha smooth muscle actin
host	Identifies the species in which the antibody was raised.	Capitalize the first letter.	Rabbit, Donkey, Mouse
isotype	Describes the antibody isotype.	Please write out any symbols.	IgG, IgG1, IgG1 kappa
clonality	Provides the clone ID (if monoclonal) or identifies the antibody as polyclonal.	List clone as provided by the manufacturer. If polyclonal, enter 'Polyclonal.'	L26, EPR5386, Polyclonal
vendor	Provides information on the source of the antibody.	Vendor name.	Cell Signaling Technology, Abcam, BioLegend
catalog_number	Provides catalog number from vendor for the source of the antibody.	Vendor catalog number.	Ab9566, sc-20060, C6198
lot_number	Allows for monitoring of lot-to-lot variation.	Number listed on antibody vial. If lot numbers are not available, please leave this field blank.	B256112, 16A5T1, KPY01191
recombinant	Classifies the antibody as recombinant or not. Recombinant antibodies (rAbs) are monoclonal antibodies which are generated in vitro using synthetic genes.	Indicate Y for yes, N for no.	Y, N
concentration_value	Provides a recommended usage in standardized units (µg/mL). If providing dilution instead, leave this field blank.	Numeric only (units standardized).	5, 2.6, 0.554
dilution	Provides a recommended dilution factor. If providing a concentration instead, leave this field blank.	1:#### (please use colon and not a slash or backslash).	1:100, 1:50, 1:2000

Field name in OMAP table	Description	Required format	Examples
conjugate	Specifies addition to the antibody (e.g., fluorophore, heavy metal, oligonucleotide) enabling detection, if applicable.	For fluorophores, please use industry standard abbreviations (e.g., AF for Alexa Fluorophore™).	AF647, PE, Oligonucleotide, 146Nd, Atto550
rrid	Allows for universal identification of an antibody (search by catalog number in the RRID Portal). If there is no entry, please register your antibody.	AB_######	AB_793620, AB_10124480
method	Specific multiplexed antibody-based imaging technique.	Provide standard abbreviation of method.	CODEX, IBEX, Cell DIVE, SIMS
tissue_preservation	Preservation technique used. If fixative other than formalin, indicate the percentage of fixative indicated (e.g., 1% or 4%).	Use a common abbreviation format (e.g., FFPE for formalin fixed paraffin embedded).	FFPE
cycle_number	Identifies the cycle number in which an antibody was either applied to the tissue or, in the case of CODEX, visualized with a fluorescent reporter. For non-cyclic methods use 1 for all cycles.	Numeric.	6, 1, 14
fluorescent_reporter	For indirect visualization (e.g., oligo-conjugated antibodies), define the fluorescent reporter utilized in the corresponding cycle. For metal or fluorophore-conjugated antibodies, please leave blank.	Fluorophore only. Please use industry standard abbreviations (e.g., AF for Alexa Fluorophore™).	AF488, Cy3, Atto550
protocol_doi	Details the protocol used to validate the antibody, including positive and negative controls and example images. To enhance reproducibility, we recommend that all protocols be made publicly available via protocols.io. Alternatively, a manuscript may be included if sufficient details on antibody validation are included in the methods section and supplementary information.	https://doi.org/… or https://dx.doi.org/…	https://dx.doi.org/10.17504/protocols.io.bqjimuke
author_orcid	Identifies the individual who validated the antibody used in the assay. See https://info.orcid.org/researchers/.	####-####-####-#### (the last digit may be X)	0000-0000-0000-0000
core_panel	Indicates whether the author considers the antibody to be part of the minimum (4-6) antibody panel for the tissue.	Indicate Y for yes (included in Core Panel), N for no.	Y, N
rationale	Why was the antibody included in the panel? What anatomical structures, cell types, or cell states of the tissue does it demonstrate? Cell state markers are highly context-specific and may be included but are not the primary focus of the OMAP effort. Please indicate whether a marker should be prioritized based on its ability to define key cell lineages and/or multiple cell types and structures.	Preferably lists anatomical structures and cell types identified by the antibody in the panel.	For CD20 in the human lymph node OMAP, “Defines B cell follicles and is an essential marker of B cells. This marker represents a prioritized target in the lymph node OMAP.

Supplementary Table 5. Information to include in the OMAP Description Document with example text.

The OMAP Description Document is a single paragraph (300 words or less) accompanying each OMAP. Required and optional fields with example text are detailed below.

Order in paragraph (Inclusion: Required or Optional)	Description	Example
1 (Required)	Include OMAP name, multiplexed imaging method, and tissue preservation method with protocols and publications DOIs (up to 3 for this field). If applicable, reference landmark study employing this OMAP.	OMAP-5 was designed for Secondary Ionization Mass Spectrometry (SIMS) imaging of fresh frozen human liver samples (https://doi.org/10.1101/2022.09.26.508878).
2 (Required)	Specify the number of antibodies included in your OMAP and the nuclear marker used for image alignment, if applicable.	The panel contains 54 antibodies and the nuclear marker Hoechst for image alignment and nuclear segmentation.
3 (Required)	Highlight overlap with ASCT+B tables and include link to organ-specific ASCT+B table.	This OMAP provides a spatial context for all anatomical structures and most cell types present in the ASCT+B lung table v1.1.
4 (Optional)	Discuss other OMAPs or multiplexed imaging studies relevant to your organ. Please include DOIs to existing OMAPs or closely related studies.	This OMAP builds from OMAP-1 (link to DOI) and additionally includes: [list markers].<br><br>The core and essential protein biomarkers detailed here overlap (>40%) with a panel developed for multiplexed imaging of kidney samples using imaging mass cytometry (IMC) (https://doi.org/10.1172/jci.insight.129477).
5 (Required)	Discuss key cells that are missing from OMAP due to the lack of suitable reagents if known.	Acinar and epsilon cells are not targeted in the existing panel due to the complexity of finding suitable antibodies.
6 (Optional)	Highlight specific application of OMAP, if any, besides mapping healthy tissues.	The inclusion of protein biomarkers DDB2 and p53 allow profiling of skin damage and aging effects.
7 (Optional)	Discuss the analytical pipeline and markers used for downstream image analysis, if applicable, with DOIs for relevant publications (up to 3 for this field).	Object-based cellular segmentation was performed using a convolutional neural network (CNN)-based approach with Mask R-CNN architecture and ResNet-50 as a backbone. As an input three channels were used: Hoechst for nuclei, CD45 as a base membrane, and composite of several other membrane markers (CD138, CD163, CD94, CD69, CD8, CD4) (https://doi.org/10.1101/2022.06.03.494716).

Order in paragraph (Inclusion: Required or Optional)	Description	Example
8 (Required)	Include links to relevant protocols, accompanying datasets, and other resources, e.g., validated antibody repository for your method (up to 3 for this field).	Additional details on sample preparation, antigen retrieval, and the Cell Dive method can be found here [protocol DOI]. Representative datasets using this OMAP can be found here [public repository DOI, e.g., https://www.doi.org/10.5281/zenodo.5244551). See (web link or DOI) for antibodies validated for this method.
9 (Required for FFPE samples)	Include the method (buffer, temperature, time, device) used for antigen retrieval and link to a detailed protocol or publication (see 8).	Following antigen retrieval (citrate buffer pH=6, heating at high pressure 114-121°C for 20 minutes), tissue autofluorescence was reduced using a photobleaching protocol (https://doi.org/10.1038/s41596-019-0206-y, modified by Derek Oldrige).
10 (Optional)	Discuss any custom conjugations or special detection methods, if applicable.	Protein targets were detected using either primary antibodies with fluorophore-labeled secondaries or fluorescent dye-conjugated antibodies. Several targets required custom conjugation to unique fluorophores by commercial suppliers or antibody conjugation kits (e.g., AF532, Thermo A20182).
11 (Required)	If applicable, note if an antibody should be placed in an earlier cycle to avoid loss of immunogenicity with cycle number.	Antibodies directed against CD106 (RRID: AB_314561) are known to perform better in cycle 1 than in later cycles.
12 (Required by December 2023)	Include a link to a PDF with Antibody Validation Reports (AVRs) for each antibody included in your OMAP.	AVRs for each antibody in OMAP can be found here (PDF).
